# Bruises in Premobile Infants: A Contested Area of Research, Policy and Practice

**DOI:** 10.1080/09503153.2022.2140132

**Published:** 2022-11-15

**Authors:** Andy Bilson, Alessandro Talia

**Keywords:** premobile, not independently mobile, non-mobile, bruise, safeguarding, section 47 enquiry, child protection investigation, physical abuse

## Abstract

This paper provides an analysis of the procedures adopted by statutory safeguarding partners throughout England in response to finding bruising in premobile infants. Against the backdrop of empirical research, we begin by challenging the view that bruising in premobile infants can be considered rare and thus suggestive of physical abuse. Then, within the procedure themselves, we point to differences in the definitions of what constitutes a premobile child, differences in the interpretation of research into bruising, and differences in how local authorities require social workers to act. We then discuss the risks involved with over-reaction to bruising in premobile children. Finally, we suggest changes to procedures that would support the appropriate use of discretion by social workers and health staff in this difficult area of practise.

## Introduction

Social workers and other front-line staff must make difficult judgments when dealing with children, particularly those aged under a year-old, who are felt to be at risk of harm. To do this effectively, they need to base their actions on a proper understanding of research into the risks that children face, and they need to have policies and procedures that help them make realistic judgments of these risks. This paper reviews guidance regarding how staff should respond when bruising is discovered in a premobile child.

Practise in this area raises difficult ethical issues. Social workers need to weigh the risk of failing to act when children may be seriously harmed against harm done by carrying out safeguarding interventions. In this paper, we propose changes to procedures that may achieve a better balance between over- and under-reaction.

## Local Authority Procedures

In 2016, Bilson (2018) reviewed local child safeguarding board procedures on bruising in premobile infants and the evidence base on which they relied. This critical analysis “found a major disjuncture between research evidence and its interpretation in guidance” (Bilson, 2018, 676). The review found that most procedures were based on the view that accidental bruises are so rare that bruising of a premobile child is likely to be abusive. Bilson (2018) showed that empirical research did not support this view, and that national guidance and local procedures exaggerated the likelihood of any bruise in a pre-mobile child being non-accidental.

Crucially, in some procedures risk was exaggerated to the extent that they suggested to bypass statutory procedures. In England, child protection investigations should only be commenced after a social work assessment has found that there is “reasonable cause to suspect” that a child “is suffering, or is likely to suffer, significant harm.” The statutory guidance for actions to be taken in safeguarding children (DfE, 2018, 36) states:

Where information gathered during an assessment (which may be very brief) results in the social worker suspecting that the child is suffering or likely to suffer significant harm, the local authority should hold a strategy discussion to enable it to decide, with other agencies, whether it must initiate enquiries under section 47 of the Children Act 1989.

This guidance requires that a social work assessment, even if very brief, should take place *before* moving to a strategy discussion (DfE 2018, 36). The strategy discussion involves relevant agencies to confirm the concerns and if so to plan any necessary action, including a formal child protection enquiry under section 47.

According to Bilson’s review, thirteen local authorities appeared not to follow this guidance. Eight of these required a strategy discussion in all cases of bruising in premobile infants, and five went further requiring a child protection enquiry in all cases. These 13 procedures thus suggested that a bruise in a premobile child provides sufficient cause to believe the child is suffering or likely to suffer significant harm without the need for a social work assessment.

Since Bilson’s review, the Children and Social Work Act 2017 transferred the duty to provide safeguarding procedures from Local Safeguarding Children Boards (LSCB) to statutory safeguarding partners (i.e. local authorities, chief officers of police, and NHS clinical commissioning groups). After a brief review of empirical studies on bruising in pre-mobile infants, this paper reviews procedures under these new arrangements.

## Research on Prevalence of Accidental Bruising

The Royal College of Paediatrics and Child Health’s (RCPCH) provides a regularly updated review of the evidence on bruising, which has been influential in shaping local procedures. The webpage introducing this review (RCPCH, 2020a) states that accidental bruising is common in children, with the exception of “non-mobile infants, where accidental bruising is rare (<1%).”^1^ The RCPCH’s updated review cites six papers focussing on the prevalence of accidental bruising in premobile children (2020 b, 10). Bilson (2018) provides a critical analysis of these six papers as well as a wider range of literature on this topic, and concludes that the evidence base is limited and contradictory (676).

Three papers cited by the RCPCH (Kemp, 2014; Carpenter, 1999; and Wedgewood, 1990) cannot support the statement that accidental bruises are rare because the sample used does not provide adequate information on the prevalence of accidental bruising in premobile children (see Table 1).

**Table 1.** Rates of accidental vs. non-accidental bruising reported in the literature.

Reference	Definition of premobile	Point Prevalence of bruising	Comments on research
Pierce et al 2016	Aged 5 months and younger	**1.3%**	US study on three sites with statistically significant differences in levels of bruising which “may be attributable to the percentage of black patients”. Also excluded “suspected abuse”. Bruising measured on a single observation.
Kemp et al 2015	Premobile: Children not yet crawling or cruising Divided into pre-rolling and rolling	*First observation* Premobile **5.3%** Pre-rolling **1.3%** Rolling **10.9%**	Observed children up to 12 times. It found 5.3% premobile infants had a bruise on 1^st^ observation and 6.7% per observation (68/1010). This is split into pre-rolling babies 1.3% were bruised on a first observation and 2.2% per observation and babies who could roll where 10.9% of babies had a bruise on a first observation and 9.8% per observation. 36/133 (27.1%) babies bruised over average 7.6 observations whilst premobile. Premobile children were aged 0-11 months and early mobile who had bruises on 45.6% of observations were aged 4–18 months.
Kemp et al 2014	Premobile: Rolling or sitting	**Not Applicable**	All children who had been referred for suspected abuse and thus cannot provide information on accidental bruising.
Sugar 1999	Pre-cruisers No upright ambulation	**2.2%** Aged under 6 months **0.6%**	US study excludes suspected abuse and bruises due to medical conditions. Statistically significantly lower rates of bruises in African American but no analysis of rate of pre-cruisers with bruises by ethnicity. Many other exclusions from the sample due to missing data. Single observation
Carpenter 1999	Aged over 6 months & ambulance no better than sits	**4.0%**	No children under 6 months Single observation
Wedgewood 1990	Not yet Cruising	**0.0%**	Sample size of 11 pre-cruisers too low to detect accidental bruises Single observation

Two of the three remaining papers are US studies (Sugar et al, 1999; Pierce et al, 2016). Bilson (2018) identified significant limitations in both. First, Sugar et al.’s study made decisions about exclusion of children from the sample that were likely to reduce the number of children with a bruise (Bilson, 2016). Namely, they excluded children “whose development appeared too advanced for their ages” (Sugar et al 1999, 401) and infants with bruises related to a medical condition. In a study that requires accurate identification of small numbers of bruised babies, any of these exclusion criteria may have led to underestimate the proportion of premobile children with a bruise that are not associated with physical abuse.

Second, both Sugar et al.’s and Pierce et al.’s studies found unexplained variations in bruising between White and African American children. In particular, Pierce et al.’s study (2016) considered attendees at three pediatric emergency departments and found that “bruising varied significantly among the three study sites” (2016, 4). These differences were attributed by the authors to “the percentage of Black patients presenting to each pediatric ED” (Pierce et al, 2016, 7), though no data was collected on the children’s ethnicity and other causes were not ruled out. Whilst there is a higher proportion of white persons and lower proportion of African Americans in San Diego (65.1% and 6.4% respectively) than in Cincinnati (50.7% white and 42.3% African American, US Census Bureau, 2021), differences in ethnicity of this magnitude could explain only a small proportion of the nearly 2.4 times higher rate of bruises found in San Diego than in Cincinnati if the detection rates of bruising was similar to that in Sugar et al’s study.

The findings on numbers of bruises by age and mobility was not disaggregated by ethnicity in either of these studies, thus the overall figure provided will significantly underestimate the number of accidental bruises found in white premobile children. It cannot be determined whether the lower number of bruises in non-White children in both these studies was because of lower detection or reporting rates of bruises; because non-White children suffer fewer accidental bruises; or because non-White children with accidental bruises were less likely to be taken to the medical facilities, perhaps because parents in these groups were concerned that child protection interventions would ensue.

The remaining study, Kemp et al (2015), recruited parents in the UK to make weekly observations of their child for up to 12 weeks. They recorded whether the child had a bruise and the current mobility of their child. To check the accuracy of the parent’s observations, a random sample of parents was visited unannounced by a qualified member of the research team. These visits confirmed the parent’s observation of the number and location of bruises in every case, making it unlikely that differences in observation rates were due to parental misidentification of bruises. This study had a smaller sample than the two US studies, covering 1010 data collections points on 133 premobile infants. The study checked that bruising was accidental, saying:

The explanations given for the bruises, where available, were compatible with the bruise sustained. In the few cases where bruise pattern was deemed unusual, they were independently reviewed by a child protection team and abuse was excluded. (Kemp et al, 2015, 430)

Kemp’s study found that 5.3% of infants not yet crawling or cruising had accidental bruises on the first observation, the best comparator to the studies having a single observation. Within this group of premobile infants, 10.9% of those who could roll had a bruise on a first observation compared to 1.3% of those who could not roll. The research did not provide findings by the child’s age, but it reported that premobile children were aged 0–11 months and early mobile infants, who were crawling or cruising, were aged 4–18 months. These figures differ from those reported in the US studies, as they indicate that some children aged under six months old were early mobile and had high rates of bruises.

Table 1 summarises the findings of the studies discussed above, along with the definition of “premobile” used by each. Whilst Pierce et al found that 1.3% of the premobile infants included in their sample had accidental bruises and Wedgewood’s study, which included only 11 premobile children, found that none of them did, the other studies cited by the RCPCH had significantly higher rates of accidental bruising (i.e. up to 5.3%).

### Is Accidental Bruising in Premobile Infants Rare?

From this research, there seems to be no valid basis for the RCPCH’s statement that only between 0 and 1.3% of premobile infants have a bruise, or for the statement on its website that <1% have a bruise and that accidental bruising is therefore rare. According to Kemp et al’s research, infants who have learnt to roll but are otherwise pre-mobile have a bruise on one in ten observations and pre-rolling infants have an accidental bruise in one in every 45 inspections. Following publicity on Bilson’s 2018 criticism of the RCPCH’s interpretation of this research, the RCPCH (2018, 1) wrote to all designated safeguarding doctors, citing the rates reported by Kemp and thus implicitly contradicting the statement that bruising in pre-mobile children is rare^2^.

Additionally, it is important to consider that all studies based on a single observation measure point prevalence, not period prevalence. The probability of an infant having bruising at any time whilst they are premobile, the period prevalence, is higher than that of displaying bruising on a single observation (Lux, 2000). Kemp et al’s study (2015, 428) found that 36 (27.1%) of 133 premobile infants had a bruise recorded over an average of 7.6 weekly observations. Thus, many premobile infants are likely to have an accidental bruise during the period of up to 11 months at this stage of development. This is significant because when a premobile child attends a nursery, is placed with a child minder, or is regularly observed by a social worker, the likelihood of an accidental bruise being found is high.

Further, in its discussion the RCPCH does not provide a definition of which children are premobile, which is a problem given the very different definitions that have been used in research. Whilst the evidence review states that bruises should not be interpreted in isolation and they should be understood in the context of “medical and social history, developmental stage, explanation given, full clinical examination and relevant investigations” (RCPCH, 2020b section 1.4), the paper’s key evidence statement is that bruising in children who are not independently mobile is suggestive of physical abuse because accidental bruising is rare.

## Are Bruises in Premobile Infants Suggestive of Physical Child Abuse?

To consider whether bruising in a premobile child is suggestive of physical abuse, we can compare the rate of accidental bruising with that of child physical abuse. Taking the most conservative estimate of the rate of accidental bruising, Sugar et al’s rate of 0.6% of children under 6 months, there would be around 1,960 accidentally bruised infants under six-month-old on any one day (based on the 2019 Office for National Statistics estimate of 653,467 children aged 0 on 30^th^ June 2017 in England). The best indicator of the number of children under 6-months-old being physically abused in one year in England is the 410 children this age who start a child protection plan under the category of physical abuse in 2016–17 (data from the first author’s freedom of information request to the Department for Education). This represents an average of 1.1 child protection plans starting per day and includes many children where the physical abuse did not involve bruising (e.g. scalds and some fractures). Even if the rate of child protection plans is a substantial underestimate of the number of infants harmed, it will remain many times more likely that a bruise on an infant will not be suggestive of abuse.

Some of the concerns about bruising in premobile children arise from the view that it is said to be “a sentinel injury”, that is, a reliable sign that physical abuse is ongoing or is likely to happen in the near future. For example, the RCPCH review claims that bruising is “widely reported” (RCPCH, 2020b, 10) as a sentinel injury. Whilst there are a few empirical studies to support the view that a significant minority of cases of physical abuse in premobile children are preceded by evidence of bruising (Pierce, et al., 2017; Sheets, Leach, Koszewski, Lessmeier, Nugent, & Simpson, 2013; Ruiz-Maldonado, Johnson, Sabo, Sheets, & Laskey, 2021), the reverse (i.e. that bruising in premobile children is a reliable indicator of ongoing or future physical abuse) has not been demonstrated. In particular, Kemp et al’s finding that 27% of premobile infants had a bruise over an average of 7.6 weekly observations suggests that many children will have an accidental bruise at some point during this stage of development. This challenges the idea that bruises in general are reliable indicators of future harm.

## Methods

In December 2020, on-line procedures for English local authorities were collected and analysed. Procedures on bruising in premobile infants were identified for 148 of the 152 local authorities. In total, there were 53 distinct procedure documents, as some local authorities were members of a consortium providing a shared procedure. In most local authorities (113) there was a stand-alone protocol on bruising of premobile infants, whist the other 35 (including 29 London authorities) mentioned bruising in premobile children relatively briefly in their main procedure document.

Content analysis of these 53 procedures (Stemler, 2001) was undertaken. As in Bilson’s study, the analysis focussed on the actions required of staff, the rationale for these actions, the definitions used to say when a child is considered premobile, and the research base cited. An Excel spreadsheet was used to create a database of the analysis and to produce numerical statistics. **Ethical approval was not required as the study consisted of a review of literature and procedure documents containing no personal information, which were freely available in the public domain.**

A consultation on the draft paper was undertaken, **as a reflective tool during the writing up,** with four sets of parents who had been subject of investigations and, in one case, had a child removed because of investigations triggered by a bruise in a premobile child. None of these parents were found to have **harmed** their child.

## Findings

### Rationale

The local authorities with a separate protocol reported the rationale that accidental bruising is very rare and that any bruise in a premobile infant is thus “highly predictive” of non-accidental injury (18 protocols). Thirty-five protocols said specifically that serious case reviews showed that staff had underestimated the significance of bruising and this had led to more serious injuries being missed. In most cases this rationale was used to justify removing the discretion of front-line staff to make judgements and making mandatory referral to children’s services, the involvement of paediatricians and in some cases child protection interventions discussed below.

The protocols focussed on studies of prevalence that were based on a single observation. They did not mention that the rate of accidental bruises likely to be found when a child is observed over time would be higher. According to Kemp et al, more than one in four premobile babies had a bruise in an average of 7 to eight weekly observations. The likelihood of identifying an accidental bruise is high, for example, if a child is in a nursery, on a child protection plan, or where a parent had voluntarily approached the referrer because of concern about a bruise. In such cases staff need to carefully assess the situation, whilst being open to a range of explanations.

### Actions to Be Taken by Staff

Most procedures mandated referral of all premobile infants with bruises to children’s social care and called for a paediatric assessment. These actions were to be undertaken immediately and police to be called if parents refused paediatric assessment. In 28 local authorities all referrals that a premobile child has a bruise automatically lead to a strategy discussion without first carrying out a social work assessment. These policies thus assume that any bruise in a premobile child is sufficient to indicate that a child is suffering or likely to suffer significant harm. They made statements such as:

Following a referral being made, a Strategy Discussion/Meeting will be held and a multi-agency decision made if an enquiry under S47 of the children act 1989 is needed to determine if the baby has suffered harm. (Bradford, Calderdale, Kirklees and Wakefield)

Seven further local authorities (Bedford Borough, Central Bedfordshire, Luton, North Lincolnshire, Sunderland, Kingston upon Thames, Richmond) appeared to require all premobile children with a bruise to be investigated under section 47 of the 1989 Children Act, for example saying:

Innocent bruising in premobile infants is rare. It is the responsibility of the strategy group members undertaking a Section 47 child protection investigation to decide whether bruising is consistent with an innocent cause or not. (Kingston and Richmond Safeguarding Partnership)

Table 2 shows how these actions relate to the protocol’s definition of when a child is premobile.

**Table 2.** Definitions used in English procedures.

Type of definition of premobile	Example definition	Number of local authorities	Protocol requires section 47 inquiry	Protocol requires strategy meeting
No definition		35		
By mobility Rolling children are treated as mobile	A baby **who is not yet rolling**, crawling, bottom shuffling, pulling to stand, cruising or walking independently. (HIPS safeguarding manual)	12		5
By mobility rolling children are treated as premobile	A baby who is not crawling, bottom shuffling, pulling to stand, cruising or walking independently. … **Babies who can roll or sit independently are classed as non-mobile.** (South West Child Protection Procedures)	29		11
All aged under 6 months and older premobile	A child who is not yet crawling, bottom shuffling, pulling to stand, cruising or walking independently, this includes all children **under the age of six months.** Please note however that **some babies can roll from a very early age and this does not constitute self-mobility**.	72	7	12

### Definitions of Premobile in English Procedures

Across the protocols, there was no consistent definition of when an infant is premobile. Table 2 shows the different types of definitions and the number of local authorities using each. Only one of the 35 local authorities where there was no separate protocol had a definition of premobile, whilst one stand-alone protocol had no definition. Procedures covering 72 local authorities said all children under 6 months of age should be considered premobile. Twenty-nine of the 41 protocols that defined mobility by developmental stage alone classified rolling children as premobile.

### Research Base

In contrast to the review of research above, many protocols claimed a strong research base for their policies:

There is a substantial and well-founded research base on the significance of bruising in children (Kingston and Richmond, 4)

Many of these protocols made statements that contradicted research findings. For example, Kemp’s research identified the following reasons for accidental bruises:

in children who were not yet able to roll over. The cause, when reported, included bumping into mother’s tooth, falling asleep on a dummy, banging themselves with a fist or rattle and a toy that was dropped on one baby …[in] children who could roll over but were not yet crawling … causes included 12 children who had fallen or toppled over, 7 rolled into something, 4 banged into an object and 6 hit themselves with an object.

23 protocols directly contradicted these statements by saying for example:

Infants **do not** bruise themselves by lying on a dummy or banging themselves with rattles and other infant toys or by flopping forwards and banging their heads against their parents’ faces. (Sheffield)

27 local authorities said bruises were found in less than 1% of premobile infants when their definitions covered children which the research showed to have higher rates, others miscited research. In Suffolk, for example, the definition of premobile children included both rolling and non-rolling infants, where Kemp et al found accidental bruises at a rate of 5.3%. However, this protocol only cited the rate of bruising in children who cannot roll:

Bruising in an infant who has no independent mobility is very uncommon - Kemp (2015) has found that 2.2% of non-mobile babies will have bruises. (Suffolk)

## Discussion

This review of procedures on bruising in premobile infants shows that there are differences in the definitions of what constitutes a premobile child, differences in the interpretation of research into bruising, and differences in how local authorities require social workers and front-line staff to act. Many procedures overestimate the likelihood that bruising is non-accidental, if we compare them to research evidence. The belief that an isolated bruise is likely to be non-accidental can lead not just to an investigation but also to the belief that the stronger criteria for emergency protection under section 44 of the Children Act 1989 are met. This is what happened to one of the parents consulted for this article, a parent whose child was removed solely because of a small bruise on its arm (see Dugan 2021).

The consultees stressed that the harm done by such over-reaction should be recognised and that an investigation alone can lead to long-term harm to parents and children. Parents remained angry and fearful of further interventions and were concerned that the investigation remained permanently on their record. One father worked in social care and was concerned that the investigation could affect his career, even though no harm was found to his child. A wide range of evidence shows that parents feel shamed, punished, powerless, and suffering injustice (e.g. Clapton, 2020; Davies, 2011; Dominelli *et al*., 2011; Smithson & Gibson, 2017) and that this impacts the whole family. In Clapton’s (2020, 18) on-line survey, parents also reported direct impacts on finances and relationships:

… the survey has uncovered worlds where considerable amounts of time must be taken off work with the prospect of losing a job or a detriment to a career, lost partners, lost friends…, the inception of poor school–family relations…, neighbour hostility … and damage to members of wider family.

Our consultees were also concerned about the risks of iatrogenic harm done to their children through exposing them to a large number of x-ray examinations. A skeletal survey for suspected child abuse has a risk of exposure-induced cancer death of one in 20,000 for a female and one in 50,000 for a male (Berger et al 2016, 310). There would be a higher risk with the addition of a CT head scan and a further increase for all of this to be repeated 10 to 14 days later as required in the case of, for example, the procedure in Surrey. There has to be strong justification to expose children to even these low risks of a cancer-induced death. Consultees said they had felt unable to assert their right to refuse x-rays, as they feared this would be interpreted as a proof they had something to hide. One of the consultee’s children was only spared a second batch of x-rays when the parents involved legal representatives at considerable expense.

### Comparison with the Earlier Survey

Since Bilson’s earlier survey, the number of local authorities with procedures relating to premobile infant’s bruising has increased from 91 to 148. As in the earlier survey, most procedures mandated referral of all premobile infants with bruises to children’s social care and called for a paediatric assessment. The number of procedures mandating a strategy meeting or section 47 child protection investigation had increased to 35 local authorities from 13 in 2016, with seven requiring section 47 inquiries and 28 requiring a strategy meeting. The concern about staff underestimating the seriousness of bruising in infants seems to have led to an exaggeration of risk in these procedures and diminution in discretion for front-line staff, with the potential for harmful over-reaction. Interestingly, of the five local authorities that mandated a section 47 in all cases of bruises in 2016, only Sunderland still did so.

### Why These Policies Should Be Changed

Most policies reviewed in this paper exaggerate the likelihood that a bruise is non-accidental. This appears to be based on a one-sided analysis of risk that focusses on not ‘missing’ a child at risk of serious harm. This causes a number of problems. The characterisation of accidental bruises as ‘extremely rare’ reduces the capacity of staff to make good decisions by properly understanding risk. It orients them to seeing any bruise as non-accidental. It reduces curiosity and exploration of alternative explanations. It leads to interventions, including child protection investigations and taking children into care, which can themselves harm children and families. Combined with the effects of limited resources and capacity of social work teams, and a basic lack of training around child development, these policies can negatively affect the judgement of paediatricians, social workers and other professionals. For example, commenting on his examination of x-rays in which a fracture was misdiagnosed following a skeletal survey triggered by two small bruises on the cheek of a premobile infant, Dr Oystein Olsen, a consultant paediatric radiologist at Great Ormond Street Hospital in London, said that the detection of abnormality should be independent of any preconception held by the radiologist:

Violating this principle would be detrimental to the entire foundation of the discipline since all current knowledge about the accuracy of diagnostic findings assume a non-biased approach. (cited in Dugan and Calver, 2021)

Finally, where an assessment takes place, most policy documents reviewed in this article call for comparing the clinical presentation with parental explanations. To our knowledge, however, there is no research into the accuracy of explanations of bruises given by parents, or about the meaning of their inability to provide explanations, or to change their explanations. In theory, a credible explanation of a significant injury should be internally coherent and consistent with the type, location, and severity of the child’s injury. Yet in the inventory of causes of injuries resulting in a bruise in premobile babies listed by Kemp et al, many may have happened in the absence of the parent, or without the parent immediately noticing (and thus later remembering) their significance. For example, falling asleep on a dummy, banging themselves with a fist or rattle, or, in rolling babies, rolling into something or banging into an object may happen in the crib or while the parent’s attention is elsewhere. Whilst under the threat of removal of their baby, parents who do not know the cause of the bruise are likely to search for a possible explanation or explanations which, if rejected, may raise groundless suspicions that they are lying or covering up. More research to support better decision making in this area is necessary.

### What Should a Procedure Say?

Safeguarding partners should review their procedures on bruising in premobile infants to make sure that they are consistent with both the evidence that bruises are often accidental and current national guidance. Practitioners should be encouraged to be curious and explore together with the parent what might have happened, whether there is any evidence of a medical condition that could have caused or contributed to the bruising, or another explanation for the presentation.

Kemp et al’s (2016) research shows that one in ten infants who can roll have a bruise on any observation. Further, before rolling 1.2% of infants had an accidental bruise, so even at this stage the chances of seeing an accidental bruise are likely to substantially outnumber non-accidental bruises. The procedure should thus limit the definition of premobile to being unable to roll and ask staff to consider whether child abuse may have caused the bruising, whilst recognising that, even in non-rolling children, the most conservative estimates indicate that accidental bruising is more likely to occur than non-accidental bruising. It is clear that simply having a bruise does not constitute likelihood of significant harm, so policies should not mandate section 47 inquiries or strategy meetings in every case.

According to Working Together, the basis for a referral is concerns about the child’s welfare or concern about significant harm. Procedures should thus require consideration of whether there is a basis for such concerns and not require referral because of a bruise alone. In all cases, it would be prudent to check records to see if the child is on a child protection plan or if there are concerns within health or social care about the child and their family, which should involve discussion with colleagues in health and social care. The child should be seen by a health professional able to assess the nature and presentation of the bruise, any associated injuries, the developmental stage of the child, and whether there may be a medical condition contributing to the bruising. When there are concerns about the adequacy of the explanation or the presentation of the family, a referral to children’s services would be in order.

## Conclusion

Most of the procedures exaggerated the risk that a bruise in a premobile child is non-accidental and mandated a referral to children’s services and a paediatrician for all bruised infants. Some go even further to mandate a child protection investigation. According to the empirical literature, there is little basis to consider the majority of bruises found in premobile children as non-accidental. Thus, there seems to be little evidence for drawing quick conclusions about risk for significant harm on the sole basis of spotting a bruise in a premobile infant. There is no justification for policies, often in adjacent local authorities, to require such different actions to be taken on bruises. Policies need to be more strongly based on research evidence and make appropriate recommendations to support front line staff to make better judgements, allowing them to build relationships rather than develop suspicion.

## Footnotes

1 The RCPH evidence review itself cites two different figures, first saying that the prevalence rate of accidental bruising in premobile infants ranges between 0 and 1.3% (RCPCH 2020b, p. 3), and then citing, three pages later, the range 0.6-1.3%.

2 Of note, the European union defines a condition as rare if it affects 1 in 2000 people in the relevant population (European Commission (EC) regulation # 141/2000).
